# The Value of Illustrations in the Lecture-Room

**Published:** 1892-11

**Authors:** L. D. McIntosh


### THE VALUE OF ILLUSTRATIONS IN THE LECTURE-ROOM.

BY L. D. M'INTOSH, M.D., D.D.S.

THE object of this paper is to point out to those who are interested in the work of the lecture-room what we believe to be one of the best methods of illustrating the various branches taught. I think I am safe in making the assertion that teachers in all departments of study see the necessity of illustrations.

Abstract teaching alone, either by lectures or recitation, fails to produce as permanent an impression on the student as is accomplished by illustrations. For example, we may give a description of a city, or some place we are familiar with, to a class of students, and afterwards ask them to explain the same; we find that no two understand it as it was given. If views had accompanied the description of the place, showing the streets, buildings, etc., each student would have formed almost as correct an idea as though he had visited it. We see how attractive and instructive the public lecturer can make his subject by using views of the scenes he portrays. It has become a necessity for him, if he desires to describe his travels, to illustrate them. The public demand this, and enforce their demand by their absence when no illustrations are shown, and, on the other hand, their approval by the large audience present when his lectures point to life-like scenes. The following shows the value of these and the permanent impression produced on an audience:

Some twelve years ago I gave a series of popular illustrated lectures in Chicago. One was entitled "Microscopic World." In the course of the lecture a description was given of the structure of a human lung, its capillary net-work, congestion, etc., using a microscopic section projected on a screen, so that it could be plainly seen by all of the audience.

Many of the persons present afterwards referred to the illustrations, that, although they had studied the subject before, this was the first time it was clear to them. Only last winter a person who was present at this lecture referred to the lecture: he said, "I am just recovering from a severe cold which came near resulting in congestion of the lungs. I imagined I could see the fine net-work of blood-vessels, in which the blood had ceased to flow, and I knew the proper thing to do was to cause it to circulate. I remembered what you said about counter-irritation and application of heat to bring the blood to the surface of the body and thus relieve the congested lung. I produced free perspiration, applied mustard-plasters freely, and was relieved in a short time."

This simply shows the impression produced by the illustrations. We can all remember the lasting effect made on us when young by the experiments of the itinerant scientific lecturer. I think my love for scientific study was awakened by listening to an illustrated lecture on natural philosophy when not more than ten years of age. It was not the words or the title that I remember, but the experiments, which seemed very wonderful to me, and are as vivid to-day as though I saw them but yesterday.

Some ten years ago I attended a medical society in a neighboring State. An evening was devoted to microscopic illustration, using a projection microscope. The public was invited, with the idea to entertain and at the same time show the scientific side of the study of medicine. A large audience was present, and all seemed interested. The work of that evening passed from my mind. Some four or five years afterwards a physician called on me; in his conversation he inquired if I remembered giving microscopic illustrations and assisting Dr. A. at a certain State medical meeting. I

told him I did. He said he was present, though not a physician at that time, and those illustrations seemed very wonderful to him. He said until this time he had looked on the profession of medicine indifferently, but what he saw that evening proved the first incentive to commence its study.

I am often chagrined and disappointed, after carefully writing a description of a scientific instrument and the directions for its use, to find the purchaser has either failed to comprehend them, or they do not give the idea clearly. A well-posted physician not long since obtained an instrument for electrical measurement. After several unsuccessful attempts to use it, he wrote me of his failure. He afterwards brought the instrument, and we placed it in proper position with connections, and it worked all right. There was one point in the directions which he did not interpret as it was intended it should be, therefore his failure; but when he saw it connected and used, the directions were plain to him.

The use of every scientific instrument and process, to be clearly understood, should be demonstrated and illustrated. Unless illustrations accompany the description, the idea is only partially comprehended. All descriptions in applications for patents must be minutely illustrated in detail, and even then expert examiners are often obliged to obtain models to aid them. If illustrations are so necessary to aid experts in comprehending descriptions in a special line, how much more does the student need them in the lecture to which he listens!

In nearly all our schools, from the primary department to the highest grades, the teachers see the necessity of illustrating their work as much as possible, and are constantly devising apparatus to do this more perfectly. The departments of Chemistry, Physiology, Anatomy, Materia Medica, Microscopy, Histology and Pathology, Operative and Prosthetic Dentistry, in fact, all scientific studies, require illustrations. Yet, with all that is being done, we believe this work is in its infancy. If there were greater effort to make a subject plain, the description cut short, given in few words and to the point, the student would retain what he sees and hears. The scientist, and even the man confined to the office, have caught the spirit of illustration. When they take their vacation, a Kodak camera is their constant companion. By its aid they bring back pictures of the country they have visited, to show their friends. Even the clergyman finds he can interest his hearers by giving an illustrated sermon, and fill the seats that would otherwise be vacant.

I believe there is no way of illustrating as easily, clearly, and cheaply the science of medicine and dentistry as by projection. We have at our command very perfect apparatus for this line of work; we can use diagrams, photo-micrographs, micro-photographs, microscopic specimens, opaque objects, chemical reaction, crystallization, polarization, etc., to large classes as easily as to one person. To make this point clear I will give you a few illustrations. Suppose my subject for consideration to-day is the circulation of the blood. Here we present to the class a diagrammatic representation of the systemic circulation, and point out its course through the vena cava into the right auricle, to the right ventricle, through the pulmonary artery to the lungs, through the capillary net-work surrounding the air-cells, back through the pulmonary vein to the left auricle, into the left ventricle, through the aorta, and through its circulatory journey. Let us now project a microscopic section of the lungs. Here we see the ultimate capillary net-work surrounding the air-cells, connective tissue, etc.; with other specimens we could show the epithelial lining, in fact, follow out all the detail and minutiæ on the screen perfectly as though we had our specimens on the stage of a microscope; and we have this advantage: we can describe it to all the class as easily as to one member, and can point out any particular structure, which we cannot do when we use the ordinary instrument. We will take another example for illustration—a diagram of the tooth, showing the enamel, dentine, etc.—and follow with a microscopic specimen. This we can see on the screen almost as plainly as with a diagram. With these illustrations the way is paved for intelligent work with the microscope in the laboratory. When a student mounts a tooth section and places it under the microscope, he sees a familiar object,—just what he has seen by the aid of projection and heard described, even to the color and delicate tints of shade. Had the lecture description been given on a blackboard instead of by the aid of actual specimens projected on the screen, he would not have recognized them when he came to the actual specimen under the microscope. We will refer to another illustration that can be made very instructive and interesting to a class,—namely, the circulation of the capillary network of a small fish or foot of a frog. Last winter I projected by the aid of the sunlight the circulation in the foot of a frog before a class. Capillaries were shown from one-half to two-thirds of an inch in diameter; the red blood-corpuscles could be plainly seen coursing through them, with now and then a white one in the field. When the web of the foot was pricked with a needle, the vessels congested round the wound, and the wounded end of one capillary projected into the opening, which appeared about three inches in diameter; the blood-corpuscles were ejected in the stream with each impulse of the heart; the circulation became slower and slower until the congestion was complete. The foot was released, rubbed to establish the circulation, and after a short time placed again under the field of the microscope. The circulation was now seen over the entire field, except the wounded part. In this short hour we had seen the circulation in the capillaries, the migration of white corpuscles, congestion, bleeding capillary, showing the impulse of the heart extending through the small vessels and the re-establishing of the circulation by massage after it had ceased. Could any description given have made the same vivid impression on those students as the illustration described ?

We might continue these illustrations by projecting specimens of every tissue of the body, but we think what has been shown will demonstrate our point. The anatomist can use photographic transparencies of any part of the body and gain in size and accuracy over the use of charts and drawings, as the object can be greatly magnified and made plain to a large class.

The chemist can use this method to advantage in the lecture-room by projecting reactions, crystallization, electrolytic action, etc., and thus pave the way for the student to begin his laboratory work with a clear idea of what he is to do.

The physiologist is dependent on illustrations to make his lectures clear, and is obliged to refer to anatomical charts and microscopic specimens. The teacher of pathology is equally interested in illustrating his work.

This method of projection is not confined to transparent bodies. Opaque bodies can as well be magnified and projected on a screen. The principle is not new, but its application to a projecting microscope we believe is. Some two years ago a scientist desired me to make for him a microscope to project objects by the aid of oblique illuminations. In constructing the instrument I found that with a little modification it could be made so as to project opaque objects. Without taking time to describe it, I will give a few illustrations of its use.

We will place a tooth on the stage, and by the aid of reflection project a clearly-defined image on the screen. In this manner any small opaque objects can be magnified and plainly shown to a class. Malformations of teeth and root, pulp-nodules, fissures, etc., can be shown by making transverse and longitudinal sections, and any small instrument or mechanical device can also be seen in this way. It is believed the teacher of operative dentistry will find in the projection of opaque objects a new field of illustration.

This method is in its infancy. There is no want of apparatus, or of light for illumination, but there is no complete list of objects, either photographic or microscopic. Each teacher is at work in his own way and uses such specimens as are within reach.

We have text-books profusely illustrated and systematically arranged. Now, what is needed is photo-diagrams, photographic transparencies, and typical microscopic slides, so that the text-books and lectures can be fully shown on the screens before classes. Drs. Sudduth, Andrews, Hays, and others have done much in this direction, each in his line of work. Others are engaged in their specialties. We trust the time is not far distant when typical objects properly arranged in sets, both photographic and microscopic, can be as easily obtained as text-books on the various subjects. The teacher will then have his notes before him in his illustration. He can abridge his lectures; this being accomplished, the poor student will not feel that he is being talked to death.

